# Effect of a culturally safe student placement on students’ understanding of, and confidence with, providing culturally safe podiatry care

**DOI:** 10.1186/s13047-021-00450-2

**Published:** 2021-01-26

**Authors:** Matthew West, Sean Sadler, Fiona Hawke, Shannon E. Munteanu, Vivienne Chuter

**Affiliations:** 1 Discipline of Podiatry, University of Newcastle, Ourimbah, NSW 2258 Australia; 2 Discipline of Podiatry, School of Allied Health, Human Services and Sport, La Trobe University, Melbourne, Victoria 3086 Australia; 3 La Trobe Sport and Exercise Medicine Research Centre, School of Allied Health, Human Services and Sport, La Trobe University, Melbourne, Victoria 3086 Australia; 4 Priority Research Centre for Physical Activity and Nutrition, University of Newcastle, Newcastle, NSW 2308 Australia

**Keywords:** Student placement, Podiatry, Cultural capability, Aboriginal and Torres Strait Islander Peoples, Survey

## Abstract

**Background:**

For university-based podiatry education there are little data available documenting the delivery method and impact of Aboriginal and Torres Strait Islander health curricula or the use of, and outcomes from, immersive clinical placements generally or specific to podiatry practice. Therefore, the primary aim of this study was to evaluate the effect of undertaking clinical placement in a culturally safe podiatry service for Aboriginal and Torres Strait Islander Peoples on podiatry students’ understanding of, and confidence with, providing culturally safe podiatry care.

**Methods:**

Final year University of Newcastle undergraduate podiatry students attending a culturally safe Aboriginal and Torres Strait Islander student clinic at a local hospital were purposively recruited to participate. Students completed a custom-made and pilot-tested cultural awareness and capability survey before and after placement. Survey domains were determined from a principle component analysis. The Wilcoxon Signed Rank test was used to compare pre-placement scores on each domain of the survey to the post-placements scores. Effect sizes were calculated and interpreted as small (0.1–0.29), medium (0.3–0.49), and large (≥0.5).

**Results:**

This study recruited 58 final year University of Newcastle podiatry students to complete baseline and follow-up surveys. For survey domain 1 (level of understanding of power relationships), domain 2 (level of understanding of the interrelationship between culture and self-perceived health), domain 3 (level of understanding of the importance of culture in clinical practice and access to health care), and domain 4 (level of confidence with providing culturally safe care) a statistically significant (*p* < 0.05) increase in scores was recorded post-placement. The effect sizes were medium to large.

**Conclusion:**

This study demonstrated that an immersive student placement at a culturally safe podiatry clinic significantly improved students’ understanding of, and confidence with, providing culturally appropriate care to Aboriginal and Torres Strait Islander Peoples. This study provides foundation evidence of the role that such placements have on developing students’ cultural capability in a tertiary health care setting, and will help inform future curricula development at both educational institutions and health services, as well as form the basis for ongoing research.

**Supplementary Information:**

The online version contains supplementary material available at 10.1186/s13047-021-00450-2.

## Introduction

Cultural capability in an allied health care setting has the goal of delivering culturally aware and safe care to every patient regardless of race, ethnicity, culture, or language proficiency [1, 2]. Cultural capability for health practitioners refers to the acquisition of skills, knowledge, and behaviours that have a grounding in, and respectful understanding of, Aboriginal and Torres Strait Islander Peoples’ culture and history [2, 3]. Developing the cultural capability of health practitioners is a key mechanism to overcome inequalities in health care access and is a determinant of health-related outcomes for Aboriginal and Torres Strait Islander Peoples [4]. Improved health care outcomes for Aboriginal and Torres Strait Islander Peoples requires health professionals to be both clinically and culturally capable [3].

Both institutional and practitioner biases are known to significantly contribute to the health disparities between Indigenous and non-Indigenous populations globally [5, 6]. Further to health care service design and provision influencing culturally safe and appropriate care, is the role of both institutions and organisations in providing curricula for effective cultural capability training [7]. Addressing deficits in cultural capability in health graduates is essential to preparing workforce-ready health professionals. A number of barriers to wide-ranging, continuous, and compulsory cultural capability training within both undergraduate and postgraduate health professional programs have been identified. For example, a lack of flexibility in pedagogical approaches to curriculum development and lack of resources to promote strong community engagement with existing programs have been identified [7, 8]. Addressing these barriers is important because recent research has demonstrated the quality of undergraduate curricula has a profound impact on entry-level health professional learners [9].

There is little research evaluating the effectiveness of cultural training programs for health professionals, with the small number of studies identified having demonstrated little to no benefit in improvements in knowledge and attitudes of health professionals [10]. Few studies have documented immersive approaches that promote community engagement and interaction, or specifically integrate participants in health care delivery, particularly in undergraduate student populations [10]. Internationally, there has been limited research performed across a number of health professions, with a mix of either individual or combined online and face to face sessions being evaluated, with studies finding a small positive relationship between cultural capability training and improved patient outcomes [11]. Several national studies have demonstrated some measure of success with improving students’ understanding of, and attitudes towards, Aboriginal and Torres Strait Islander health and self-perceived preparedness for practising in Indigenous communities and advocating for improved health outcomes [10, 12]. For university-based podiatry education there are little data available documenting the delivery method and impact of Aboriginal and Torres Strait Islander health curricula or the use of, and outcomes from, immersive clinical placements generally or specific to podiatry practice. While there are a number of podiatry services, resources, and educational tools for Aboriginal and Torres Strait Islander Peoples in Australia [13], such programs that do include podiatry students do so as part of a large allied health professional team with no in-depth evaluation of the experience from the perspective of podiatry students [14], or the role of such programs in podiatry student cultural capability training.

Therefore, to evaluate the effect of an immersive clinical placement on self-perceived cultural awareness and capability, a student placement program was developed for all final year podiatry students at the University of Newcastle, in a University-led podiatry service for Aboriginal and Torres Strait Islander Peoples. The primary aim of this study was to evaluate the effect of undertaking clinical placement in a culturally safe podiatry service for Aboriginal and Torres Strait Islander Peoples on students’ understanding of, and confidence with, providing culturally safe podiatry care.

## Methods

### Design and participants

Final year University of Newcastle undergraduate podiatry students attending a culturally safe Aboriginal and Torres Strait Islander student clinic at Wyong Hospital (located on the Central Coast, between Sydney and Newcastle, New South Wales (NSW), Australia), were purposively recruited to participate. This project was approved by the University of Newcastle Human Ethics committee (protocol number H-2018-0035) and the Aboriginal Health and Medical Research Council (1376/18). Informed consent was provided by all participants.

### Clinical placement

The clinic has been developed as a culturally safe podiatry service for the Aboriginal and Torres Strait Islander community on the Central Coast of NSW, Australia. The clinic is led by an Aboriginal Podiatrist, supported by an Aboriginal Health Worker, and provides student placements throughout both teaching semesters. The culturally safe podiatry clinic is designed to create an environment that is considerate of the spiritual, physical, social, and emotional world view of Aboriginal and Torres Strait Islander Peoples, thereby creating a clinical experience which is conducive to, and supportive of, the specific needs of this community [15]. Additionally, the approach to the management of clients within this clinic is one that recognises the importance of culture, family, and community for Aboriginal and Torres Strait Islander Peoples. For example, clinical consultations are structured around group appointments and two intake times, as opposed to a traditional individual appointment time. This collective-participation approach encourages open discussion amongst community members, assisting students in understanding lived experiences and history sharing, and creates a sense of ownership of the clinic for Aboriginal and Torres Strait Islander Peoples. The clinic is managed by the University of Newcastle, Discipline of Podiatry, and operates a weekly foot care service for Aboriginal and Torres Strait Islander Peoples. The clinic provides a range of podiatry care and health promotion education, with a focus on diabetic foot disease (DFD) and prevention education. Final year students undertake a block mode delivery (single face to face session) cultural capability training program over a whole day, delivered by an Aboriginal Podiatrist and an Aboriginal Elder from the local community before being rostered through the clinic. Students recruited to the present study were in the final year of the podiatry program in 2018 or 2019, and participated in a minimum of four one-day placements in the clinic over the first and second semesters of the academic year. During these clinic sessions, students provided podiatry assessments and management, patient education about strategies to maintain good foot health, and participated in pre- and post-appointment yarning circles with clinic clients. The first yarning circle was led by the Aboriginal Health Worker or Aboriginal Podiatrist and served as a mechanism of introducing the students to the community participants. The yarning circle post-appointment facilitated a communal and informal opportunity for culturally responsive health promotion activities and clinic feedback. Following the placement, the students completed a reflective journal relating to their experiences in the clinic. Successful completion of the placement program and associated placement reflection was a compulsory part of the clinical placement program. Students had additional opportunities to undertake voluntary placements at other related community events and outreach clinics run for Aboriginal and Torres Strait Islander Peoples throughout the year.

### Procedure

Final year undergraduate podiatry students attending the placement voluntarily completed the cultural awareness and capability survey (Cultural Capability for Podiatry Students) after their cultural capability training session but before undertaking any clinical placement, and also following the 4 days of placement over the two semesters. The second survey completion was approximately 7 to 8 months later according to their placement roster and directly after their final one-day placement. Surveys were administered via hard copy by a researcher not involved in the supervision of the students.

### Cultural capability for podiatry students survey

A customised survey was developed to measure aspects of students’ self-perceived cultural awareness and capability. The survey was developed drawing on existing survey literature [16–20], and utilising a participatory action approach [21]. This involved a multi-stage process including initial systematic exploration and analysis of evidence and establishment of contextual relevance through review of existing literature, and Aboriginal education and podiatry education expert review [22]. Testing for feasibility, relevance, and appropriateness/comprehension of the survey was undertaken with 10 final year podiatry students. Thematic analysis of feedback provided at each stage of this process was used to refine the survey. The final version of the survey then underwent principle component analysis to identify individual questions that were similar enough to be grouped together to create domains. Scores from each question within each of the domains were summed to create a total score per domain. Identification of these domains made it possible to perform statistical analyses comparing the students’ scores per domain from before to after their placement. However, prior to principle component analysis, the suitability of survey data for factor analysis was assessed. Inspection of the correlation matrix of individual question responses at baseline revealed the presence of several coefficients above 0.3, indicating commonality between sections of the survey. Additionally, the Kaiser-Meyer-Olkin value exceeded 0.6 and the Bartlett’s Test of Sphericity reached statistical significance. All of which support the use of principle component analysis [23]. To aid interpretation of components within the survey, oblique rotation of factors using Oblimin rotation was performed. The number of components extracted was based on the Kaiser’s criterion (eigenvalue above 1) [24] and inspection of the scree plot (with components below the ‘elbow’ rejected) [25]. The internal consistency of final components was investigated using Cronbach’s alpha coefficient, with values above 0.7 representing acceptable reliability [26].

Principle component analysis revealed the presence of five components with eigenvalues exceeding 1, explaining 23.7, 21.1, 12.5, 7.4, and 6.2% of the variance respectively [26]. Inspection of the scree plot revealed a change (elbow) between components 4 and 5, therefore, four components were retained [25]. The four component solution explained a total of 64.7% of the variance. To aid in the interpretation of these four components, Oblimin rotation was performed. The rotated solution revealed substantial loading of individual variables mostly on single components. The correlation between each component was weak with values ranging from *r =* 0.02 to *r* = 0.35 (see Additional file 1 for component correlation matrix), suggesting each component is measuring different aspects of the survey. Questions from the survey that fit into each component can be found in Additional file 2. Pattern and structure matrices for the principle component analysis with Oblimin rotation of the four factor solution can be found in Table 1. Cronbach’s alpha coefficient for component 1 (0.87), component 2 (0.81), component 3 (0.79), and component 4 (0.76) exceeded 0.7, suggesting acceptable internal consistency of the domains [26] (Table 1).

**Table 1:** Pattern and structure matrices for principle component analysis with Oblimin rotation of four factor solution

Survey Question	Pattern Matrix				Structure Matrix				Communalities
	Component				Component				
	1	2	3	4	1	2	3	4	
Q15	**0.858**	0.061	− 0.088	−0.082	**0.829**	0.074	0.105	−0.010	0.707
Q16	**0.848**	−0.047	0.109	0.053	**0.879**	−0.084	0.297	0.157	0.790
Q17	**0.837**	−0.006	0.094	0.080	**0.866**	−0.052	0.278	0.169	0.765
Q14	**0.785**	0.023	0.059	−0.135	**0.784**	0.008	0.238	−0.045	0.635
Q7	−0.066	**0.873**	0.197	−0.148	−0.020	**0.822**	0.197	0.147	0.736
Q6	0.249	**0.826**	−0.187	0.064	0.231	**0.851**	−0.124	0.386	0.809
Q4	− 0.171	**0.660**	−0.065	0.136	−0.158	**0.703**	−0.099	0.351	0.545
Q5	0.327	**0.549**	−0.119	0.294	0.343	**0.658**	−0.048	0.525	0.633
Q8	−0.061	**0.530**	0.086	0.287	−0.001	**0.631**	0.071	0.465	0.474
Q12	−0.032	0.033	**0.864**	−0.033	0.156	0.035	**0.857**	−0.073	0.739
Q10	0.016	0.039	**0.783**	−0.048	0.184	0.046	**0.787**	−0.083	0.625
Q11	0.178	0.012	**0.751**	0.074	0.353	−0.027	**0.788**	0.068	0.659
Q9	−0.069	0.240	**0.728**	−0.179	0.079	−0.185	**0.721**	−0.122	0.586
Q13	0.114	0.125	**0.519**	0.250	0.254	0.028	**0.536**	0.203	0.361
Q2	−0.081	−0.061	−0.049	**0.821**	−0.004	− 0.348	−0.090	**0.835**	0.712
Q3	−0.023	−0.054	0.061	**0.794**	0.076	−0.334	0.034	**0.809**	0.660
Q1	0.001	−0.044	−0.025	**0.729**	0.073	−0.300	−0.045	**0.745**	0.557

Extraction Method: Principal Component Analysis. Rotation Method: Oblimin with Kaiser Normalization. Rotation converged in 8 iterations Note: major loadings per component are bolded

The final survey included 17 statements with respondents asked to consider how likely they are to agree or disagree with each statement on a 5-point Likert scale ranging from ‘strongly agree’ to ‘strongly disagree’ (Additional file 2). The survey covers fours domains of cultural awareness and capability: 1) *Level of understanding of power relationships; 2) Level of understanding of the interrelationship between culture and self-perceived health; 3) Level of understanding of the importance of culture in clinical practice and access to health care; and 4) Level of confidence with providing culturally safe care.*

### Statistical analysis

Survey data were entered into Microsoft Excel and then exported to the statistical packages for social sciences program for analysis (version 25.0 Chicago, Illinois, USA). Data were assessed for normality using histograms, boxplots, and the Shapiro-Wilk test with appropriate parametric or nonparametric analyses performed.

The Wilcoxon Signed Rank test was used to compare pre-placement scores on each domain of the survey to the post-placements scores. Effect sizes were calculated using Microsoft Excel and reported using the z value statistic divided by the square root of N (cases × 2) with the size of the effect (r) interpreted according to Cohen [27]: 0.1–0.29 = small effect; 0.3–0.49 = medium effect; ≥0.5 = large effect.

## Results

This study recruited 58 final year University of Newcastle podiatry students to complete baseline surveys and follow up surveys over two years (2018 and 2019). The recruited participants included 31 female students and 27 male students, all of whom completed the pre-placement cultural capability training. The minimum and maximum number of one-day placements ranged from three to five, depending on student rotation, with the mean number of sessions equalling 3.9. Additionally, 16 students attended the annual NAIDOC day (8 each in 2018 and 2019), and nine attended an outreach clinic in Wellington, NSW (3 in 2018 and 6 in 2019). The age of participants was not collected.

### Change in survey domain scores

*Domain 1: Level of understanding of power relationships (survey questions 1 to 3)*

The median score on component 4 increased from pre-placement (*Md* = 13.00/15.00) to post-placement (*Md* = 14.00/15.00), z = − 5.47, *p* < 0.001, with a large effect size (*r* = 0.51) (Table 2).

**Table 2:** Pre-placement and post-placement scores for each domain of the survey. All values are median (IQR) unless otherwise specified

Domain (number of questions, max score)	N	Pre-placement score	Post-placement score	Score change (% of pre-placement score)	Z	P	Effect size
1 (3, 15)	58	13.00 (11.00 to 14.00)	14.00 (13.00 to 14.00)	1.00 (7.69%)	−5.47	< 0.001	*r* = 0.51 (large effect)
2 (5, 25)	58	19.00 (17.00 to 20.00)	22.00 (21.00 to 23.00)	3.00 (15.79%)	−6.36	< 0.001	*r* = 0.59 (large effect)
3 (5, 25)	58	23.00 (21.00 to 24.00)	24.00 (22.00 to 25.00)	1.00 (4.35%)	−4.16	< 0.001	*r* = 0.39 (medium effect)
4 (4, 20)	58	11.00 (9.75 to 15.00)	17.00 (16.00 to 18.00)	6.00 (54.55%)	−6.58	< 0.001	*r* = 0.61 (large effect)

*Domain 2: Level of understanding of the interrelationship between culture and self-perceived health (survey questions 4 to 8)*

The median score on component 2 increased from pre-placement (*Md* = 19.00/25.00) to post-placement (*Md* = 22.00/25.00), z = − 6.36, *p* < 0.001, with a large effect size (*r* = 0.59) (Table 2).

*Domain 3: Level of understanding of the importance of culture in clinical practice and access to health care (survey questions 9 to 13)*

The median score on component 3 increased from pre-placement (*Md* = 23.00/25.00) to post-placement (*Md* = 24.00/25.00), z = − 4.16, *p* < 0.001, with a medium effect size (*r* = 0.39) (Table 2).

*Domain 4: Level of confidence with providing culturally safe care (survey questions 14 to 17)*

The median score on component 1 increased from pre-placement (*Md* = 11.00/20.00) to post-placement (*Md* = 17.00/20.00), z = − 6.58, *p* < 0.001, with a large effect size (*r* = 0.61) (Table 2).

## Discussion

The primary aim of this study was to evaluate the effect of a culturally immersive student placement on students’ understanding of Aboriginal and Torres Strait Islander health, and confidence with providing culturally safe podiatry care to a community-based population of Aboriginal and Torres Strait Islander Peoples. We found that the placement significantly improved students’ understanding of multiple aspects of cultural capability (e.g. understanding of culture, history and interrelationship with health and health care delivery), and level of confidence with providing culturally appropriate and safe podiatric care. This study provides foundation evidence of the role that such placements have on developing students’ cultural capability in a tertiary health care setting, and will help inform future curricula development at both educational institutions and health services, as well as, form the basis for ongoing research. Additionally, the student cultural capability survey developed as part of this study can be used to assist with evaluation of any future curriculum changes, from the perspective of quality assurance, as well as to provide ongoing assessment of students’ experiences in the Aboriginal and Torres Strait Islander clinic.

To the authors’ knowledge, this is the first study to evaluate the effect of an immersive student placement on undergraduate podiatry students’ self-perceived cultural capability in an Australian setting, making comparison with existing literature challenging. Existing studies typically evaluate a suite of training methods (e.g. online, lecture setting, group work, and placements), and are primarily focused on both Indigenous populations of countries other than Australia and experiences of other health professions such as medicine and nursing [28–30]. Likewise, studies within Australia have only considered the effect more broadly from the perspective of multiple allied health professions, with discipline specific evaluations (such as podiatry) lacking [10, 13]. However, findings are generally consistent with our research in that participants’ awareness, understanding, and confidence with providing culturally appropriate health care improves following exposure to culturally appropriate and safe learning. Specifically, the current study has identified that an immersive, hands-on student placement experience is an effective delivery mechanism for improving undergraduate podiatry students’ understanding of the role that culture has on Aboriginal and Torres Strait Islander Peoples’ perceptions of health and health care (domains 2 and 3). Additionally, this placement resulted in significant improvements in students’ understanding of the importance of and responsibility that they, and all health professionals have, in creating an environment that enables Aboriginal and Torres Strait Islander Peoples to feel safe and respected (domain 1).

Students’ confidence with providing culturally appropriate communication and management strategies for effective engagement with and continuity of care of Aboriginal and Torres Strait Islander Peoples were also found to significantly improve following this placement (domain 4). This ability to engage with, and ensure, Aboriginal and Torres Strait Islander Peoples form an integral part of the holistic approach to health care is a vital step in developing trust with such services. Additionally, existing literature supports this shared approach to knowledge and understanding of Aboriginal and Torres Strait Islander health needs as an integral step in developing intercultural partnerships [2, 31, 32]. These types of partnerships have been shown to be fundamental to empowering Indigenous communities to take control of their health [33], and for health services to adapt from Western models of care to service design and delivery that recognises the role of culture, family, and community in Aboriginal and Torres Strait Islander Peoples’ health [34]. This renewed approach, in which Aboriginal and Torres Strait Islander Peoples are a central part in the design and delivery of health care, may help close the gap in a number of health inequities.

Globally a number of barriers to accessing preventative care among Indigenous populations have been identified, including poor relationships with health care providers, health care providers’ lack of acceptance (or appreciation) of the role of family in care provision, and poor community engagement with available services [34–37]. The positive impact of placement in a culturally safe podiatry clinic demonstrated by the present study highlights this as a potential long-term strategy to address deficits in availability of culturally safe care, with the capacity to generate large scale positive change in the future podiatry workforce. Similarly, the capacity for translation of comparable clinical training initiatives across tertiary allied health training is significant. Lack of understanding culturally safe care delivery and practitioner bias are key barriers to engagement with, and uptake of, health care by Aboriginal and Torres Strait Islander communities [34, 36, 37]. Producing graduate clinicians considerate of the role of culture, family, and community in Aboriginal and Torres Strait Islander Peoples’ perception of their own health and health care is crucial to successfully reducing the burden of disease in this population. This shift from a deficit-focused to a strength-based approach for culturally safe podiatry care and health education is key to enabling self-determination of Aboriginal and Torres Strait Islander Peoples’ health and wellbeing [38].

The following limitations of this study should be considered when interpreting our findings. We only included a small number of undergraduate podiatry students from one Australian University who participated in a limited number of clinical sessions. The high response rate may have been influenced by the relationship between the participants and researchers. However, given that the cultural capability for podiatry students survey was administered by researchers not supervising students, and participation was voluntary, this is not likely. The survey used to evaluate the placement assessed students’ own perceptions of aspects of their cultural capability. Additionally, the changes in pre-placement scores for each domain in the survey may have been influenced by other factors besides the placement at the student podiatry clinic. For example, external placements and participation in local community events (e.g. NAIDOC), may have influenced students’ cultural capability, perhaps accounting for some of the change demonstrated in the post-placement scores on the cultural capability survey. However, these additional sessions were limited in duration (single day) or only undertaken by a small number of students, suggesting that it would be unlikely that they significantly impacted the post-placement scores on the survey. Although the changes in scores for each domain of the cultural capability survey were significant, further research is required to determine whether such changes are clinically meaningful. Assessment of the experiences of clients in the clinic did not comprise part of this study and change in specific knowledge or behaviours was not evaluated. Nevertheless, these findings provide a preliminary understanding of the effect that these types of placements have on cultural capability learning and will help inform future research. Additionally, it is important to highlight that patient-centred health-related outcomes were not evaluated as part of this study, and it is currently unknown if the same placement results in improved health related outcomes for these communities. Caution is advised when comparing this study’s findings with existing literature, or attempting to generalise to other Aboriginal and Torres Strait Islander communities, due to the culturally diverse nature, kinship, and beliefs of Indigenous Peoples across all First Nations of Australia and other countries.

## Conclusion

This study found that students, who participated in an immersive placement at a culturally safe podiatry clinic, had significant improvements in their understanding of, and confidence with, providing culturally appropriate care to Aboriginal and Torres Strait Islander Peoples. Educational institutions should consider these findings as part of the suite of methods to deliver cultural capability training to undergraduate allied health professional students. Future research should continue to assess the role of such placements on cultural capability training in larger sample sizes, investigate if such programs are transferrable to practising health professionals, as well as prospectively evaluate the impact of clinicians’ improved cultural capability on Aboriginal and Torres Islander Peoples’ engagement with health services and health-related outcomes.

## Supplementary Information

**Additional file 1.** Component correlation matrix.

**Additional file 2.** Student cultural capability questionnaire.

## Data Availability

Requests for further detail on the data collected in this study, or data sharing arrangements, can be submitted to Vivienne Chuter (Vivienne.chuter@newcastle.edu.au).

## Abbreviations

DFD: Diabetic foot disease
Md: Median
NSW: New South Wales
