# Supplementary material for: Effect of a culturally safe student placement on students’ understanding of, and confidence with, providing culturally safe podiatry care
Source: J Foot Ankle Res. 2021 Jan 26;14:9. doi: 10.1186/s13047-021-00450-2 (PMC7836510; doi:10.1186/s13047-021-00450-2)
Supplement: Supplementary file 1 — Additional file 1. Component correlation matrix. [file 13047_2021_450_MOESM1_ESM.docx]

| **Component Correlation Matrix** | | | | |
| --- | --- | --- | --- | --- |
| Component | 1 | 2 | 3 | 4 |
| 1 | 1.000 | -0.020 | 0.223 | 0.106 |
| 2 | -0.020 | 1.000 | -0.012 | -0.352 |
| 3 | 0.223 | -0.012 | 1.000 | -0.028 |
| 4 | 0.106 | -0.352 | -0.028 | 1.000 |
| Extraction Method: Principal Component Analysis.   Rotation Method: Oblimin with Kaiser Normalization. | | | | |
