# Supplementary material for: Effect of a culturally safe student placement on students’ understanding of, and confidence with, providing culturally safe podiatry care
Source: J Foot Ankle Res. 2021 Jan 26;14:9. doi: 10.1186/s13047-021-00450-2 (PMC7836510; doi:10.1186/s13047-021-00450-2)
Supplement: Supplementary file 2 — Additional file 2. Student cultural capability questionnaire. [file 13047_2021_450_MOESM2_ESM.docx]

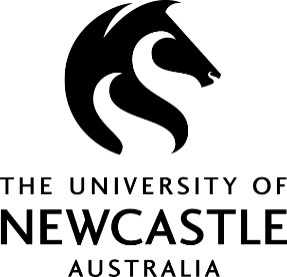


**Student Questionnaire: Improving foot health outcomes for Aboriginal Australians with diabetes**

A.Prof. Vivienne Chuter

School of Health Sciences

University of Newcastle

Health Precinct, Ourimbah Campus

Ph: 02 43494424

Fax: 02 43494538

Email: [Vivienne.Chuter@newcastle.edu.au](mailto:Vivienne.Chuter@newcastle.edu.au)

The following section contains questions relating to your views on cultural awareness and safety. For each statement please indicate your level of agreement by ticking the corresponding circle.

| For each of the following please indicate how much you agree or disagree with the statement | **Strongly Agree** | **Agree** | | | **Neutral** | | **Disagree** | | **Strongly Disagree** |  |
| --- | --- | --- | --- | --- | --- | --- | --- | --- | --- | --- |
| 1. I understand the balancing of power relationships and how this relates to the practice of health professionals. | ○ | ○ | | | ○ | | ○ | | ○ |  |
| 2. It is the responsibility of the health care provider as the power-holder to create an environment that enables people to feel safe in the presence of the health care provider. | ○ | ○ | | | ○ | | ○ | | ○ |  |
| 3. I understand the effect of racism on self-identity, and its potential impact within a health context. | ○ | ○ | | | ○ | | ○ | | ○ |  |
| 4. I understand the concept of culture and how it relates to identity for Aboriginal and Torres Strait Islander Peoples. | ○ | ○ | | | ○ | | ○ | | ○ |  |
| 5. I am knowledgeable about the health of Aboriginal and Torres Strait Islander Peoples. | ○ | ○ | | | ○ | | ○ | | ○ |  |
| 6. I am knowledgeable about Aboriginal and Torres Strait Islander Peoples history. | ○ | ○ | | | ○ | | ○ | | ○ |  |
| 7. I am knowledgeable about the continued impact of historical influences on health and wellbeing of Aboriginal and Torres Strait Islander Peoples. | ○ | ○ | | | ○ | | ○ | | ○ |  |
| 8. I know clearly what constitutes an act of prejudice in a healthcare setting. | ○ | ○ | | | ○ | | ○ | | ○ |  |
| 9. I would like to know more about Aboriginal and Torres Strait Islander culture. | ○ | | ○ | ○ | | ○ | | ○ | |  |
| 10. Being culturally aware is relevant to my clinical work. | ○ | | ○ | ○ | | ○ | | ○ | |  |
| 11. Cultural safety within hospital policies and practices improves the health outcomes for Aboriginal and Torres Strait Islander Peoples. | ○ | ○ | | | ○ | | ○ | | ○ |  |
| 12. Healthcare services should be modified to better suit the needs of Aboriginal and Torres Strait Islander. | ○ | ○ | | | ○ | | ○ | | ○ |  |
| 13. I understand the important of using culturally appropriate forms of communication when interacting with Aboriginal and Torres Strait Islander Peoples. | ○ | ○ | | | ○ | | ○ | | ○ |  |
| 14. I am confident in using culturally appropriate forms of communication when interacting with Aboriginal and Torres Strait Islander Peoples. | ○ | ○ | | | ○ | | ○ | | ○ |  |
| 15. I am confident in applying knowledge of Aboriginal and Torres Strait Islander Peoples definitions’ of health and wellbeing to clinical practice. | ○ | ○ | | | ○ | | ○ | | ○ |  |
| 16. I am confident in responding sensitively and appropriately to the needs of Aboriginal and Torres Strait Islander peoples. | ○ | ○ | | | ○ | | ○ | | ○ |  |
| 17. I am confident of providing culturally safe care to Aboriginal and Torres Strait Islander Peoples. | ○ | ○ | | | ○ | | ○ | | ○ |  |

This questionnaire has been developed from the following resources:

D'Andrea, M., Daniels, J., & Noonan, M. J. (2003). New Developments in the Assessment of Multicultural Competence: The Multicultural Awareness-Knowledge-Skills Survey--Teachers Form.

Jones, J. (2013). *The Multicultural Awareness, Skills, and Knowledge Survey: An instrument for assessing the cultural competency of pre-service teachers*: New Mexico State University.

Milne, T., Creedy, D., & West, R. (2016). Development of the Awareness of Cultural Safety Scale: A pilot study with midwifery and nursing academics. *Nurse education today, 44*, 20-25.

Ponterotito, J. G., Baluch, S., Greig, T., & Rivera, L. (1998). Development and initial score validation of the teacher multicultural altitude survey. *Educational and psychological measurement, 58*(6), 1002-1016.

Ramsden, I. (2002). *Cultural safety and nursing education in Aotearoa and Te Waipounamu.* Victoria University of Wellington New Zealand.
